# Unexpected Events Resist Directed Forgetting

**DOI:** 10.1007/s41465-026-00365-5

**Published:** 2026-06-20

**Authors:** Alex Kafkas, Aisha Mukhtar, Sadie Griffiths, Jingqiu Zhou

**Affiliations:** School of Health Sciences, Andrew Mayes Centre for Cognitive Neuroscience, University of Manchester, Oxford Road, Dover Street Building, Manchester, M13 9PL UK

**Keywords:** Expectation, Memory, Prediction-error, Novelty, Directed forgetting, Recollection

## Abstract

This study examined whether expectation violations modulate the effectiveness of directed forgetting (DF) and whether this interaction varies with emotional valence. We hypothesised that unexpectedness may confer mnemonic resilience, protecting from later forgetting. Participants first learned symbol–stimulus contingencies (rule learning), followed by an encoding phase with new emotional and neutral stimuli that were either expected or violated the learned rule. Each stimulus was accompanied by a “remember” or “forget” cue, and a subsequent recognition test assessed memory performance for expected and unexpected items under both DF conditions. Unexpected stimuli were remembered better than expected ones, particularly through recollection, and this advantage persisted even under forget instructions, indicating resistance to DF. Forget cues reduced recollection overall, but this reduction was markedly stronger for expected than for unexpected items. Emotional valence partly moderated these effects with the expectation advantage for recollection being evident for negative and neutral stimuli but not for positive ones. Nevertheless, the weakening of the DF effect for unexpected stimuli occurred irrespective of emotional valence. These findings demonstrate that prediction error enhances memory formation and diminishes the efficacy of intentional forgetting, particularly for associatively richer, recollection-based memories. The results support a view of DF as a dynamic process constrained by encoding factors such as surprise and emotional salience, with implications for theories of memory control and applications in education and memory training.

## Introduction

Human memory is both selective and limited, operating under constraints that require the prioritisation of certain information at the expense of others (Schacter, 2022). This selectivity is not arbitrary but guided by cognitive and environmental factors such as attention, emotional salience, and the fit between new information and prior expectations (Plater et al., 2023). Expectation, in particular, plays a pivotal role in shaping how events are encoded, stored, and later retrieved, yet its contribution to the formation of *long-lasting* memories that may be less susceptible to forgetting, has not been examined.

Events that conform to prior expectations are processed efficiently but often superficially, relying more on familiarity at retrieval (Kafkas & Montaldi 2018a). In contrast, unexpected events that violate predictions require greater cognitive resources, capture attention, and are encoded more vividly, increasing the likelihood of associative recollection (Frank & Kafkas 2021; Kafkas 2021; Kafkas & Montaldi 2015, 2018a, 2018b; Phelps & Sharot 2008). Recognition memory is commonly understood to reflect separable contributions of recollection and familiarity, with recollection involving retrieval of contextual or associative detail and familiarity reflecting a sense of prior occurrence in the absence of such detail (Jacoby, 1991; Yonelinas, 2002; Montaldi & Kafkas, 2024). However, not all events are encoded with equal contextual richness leading to recollection. Contemporary predictive-processing accounts propose that violations of expectation trigger prediction-error signals that recruit hippocampal and dopaminergic systems involved in learning (Frank & Kafkas 2021; Kafkas & Montaldi 2018b; Quent et al. 2021). These signals promote the formation of distinctive episodic representations, which are more likely to be encoded with richer contextual detail and later retrieved through recollection (Büchel et al., 2017; Foster & Keane, 2019; Greve et al., 2019; Reichardt et al., 2020). These memory benefits are most consistently observed in studies using contingency-based designs, in which expectations are built through recently learned associations (Frank & Kafkas 2021; Greve et al. 2019; Kafkas 2021; Kafkas and Montaldi 2018a; Quent et al. 2021).

For example, in a series of studies (Kafkas 2021; Kafkas & Montaldi 2018a; Mukhtar & Kafkas 2026), participants first learned contingencies between arbitrary symbols and stimulus categories (e.g., natural versus man-made or orange- versus purple-framed stimuli). During the encoding phase, some symbol–stimulus combinations were consistent with these learned contingencies (expected), whereas others violated them (unexpected). The findings showed that expectation at encoding influenced the type of recognition memory produced afterwards. Specifically, familiar responses were more common for expected items, while unexpected items were more likely to evoke recollection. Kafkas and Montaldi (Kafkas & Montaldi 2018a, 2018b) proposed that the reason for this memory superiority for unexpected events relates to the engagement of pattern separation mechanisms, which generate distinctive representations facilitating later retrieval even among competing memories. This enhanced distinctiveness may underlie the mnemonic advantage commonly observed for unexpected or surprising information.

Expanding this, neurocognitive models attribute these effects to distinct neuromodulatory pathways (Frank & Kafkas, 2021). According to this account, expected events are primarily encoded via cholinergic signalling within medial temporal lobe regions, whereas unexpected events engage dopaminergic–hippocampal mechanisms that promote elaborative encoding and recollection (Kafkas, 2021; Kafkas & Montaldi, 2015). Novelty and salience amplify this effect with prediction violations biasing hippocampal processing towards the formation of new, unique representations (Allegra et al., 2020), while dopaminergic signalling reinforces the consolidation of such novel inputs (Duszkiewicz et al., 2019; Kafkas & Montaldi, 2015; Yi et al., 2025). Taken together, these findings suggest that unexpected events may confer mnemonic resilience by enhancing the strength and distinctiveness of encoded representations. If violations of expectation engage neuromodulatory and attentional processes that promote elaborative encoding, they may produce memory traces that are less susceptible to subsequent forgetting.

Emotional arousal can enhance memory strength increasing the likelihood that emotional events will be retained over time (Kensinger & Fields, 2024). Such events are typically remembered more vividly and are less susceptible to intentional forgetting (Nowicka et al., 2011). However, the extent to which emotional encoding influences susceptibility to intentional forgetting remains debated (Hall et al., 2021). Emotionally arousing experiences tend to produce stronger recognition memory than neutral ones, leading to better retention overall (LaBar & Cabeza, 2006; Reisberg, 2006; Todd et al., 2012) and are often associated with vivid recollection (Phelps & Sharot, 2008). However, the precise mechanisms underlying emotion’s memory advantage remain a matter of debate (Adolphs et al., 2005; Bowen et al., 2018; Dolcos et al., 2005; Johansson et al., 2004; Pourtois et al., 2013; Reisberg, 2006; Talmi, 2013). One proposal states that emotional events engage amygdala–hippocampal interactions and catecholaminergic responses that strengthen consolidation (Hall et al., 2021; Schott et al., 2011). Consistent with the consolidation account, emotional events have been shown to engage amygdala–hippocampal interactions leading to stronger and more persistent memory traces (McGaugh, 2004). An alternative perspective highlights attentional and prioritisation processes, whereby emotionally salient stimuli capture attention and receive enhanced encoding (Mather & Sutherland, 2011). A related view proposes that emotion influences the distinctiveness and organisation of memory representations, thereby facilitating later retrieval (Kensinger, 2009). The present study does not adjudicate directly between these accounts but examines whether emotional modulation operates through enhanced encoding strength, which may in turn influence the effectiveness of directed forgetting. Consistent with these accounts, the convergence of emotional intensity and prediction error may also produce a particularly enduring form of memory. Indeed, a recent study showed that the memory benefit of unexpected events varies with emotional arousal, with negative stimuli gaining a particular advantage when they are unexpected, highly arousing, and presented within mixed-emotion lists (Mukhtar & Kafkas, 2026).

Directed forgetting (DF) paradigms provide a useful framework for examining the extent to which memory can be intentionally downregulated. DF paradigms demonstrate that individuals can intentionally reduce memory for specific information when instructed to do so (Anderson & Hulbert, 2021; Macleod, 1999). In the item-method paradigm, each item is followed by a cue to remember or forget, typically resulting in poorer memory for to-be-forgotten (TBF) than to-be-remembered (TBR) items. (Macleod, 1999). Importantly, item-method DF is generally understood as an encoding-based phenomenon, reflecting processes that operate at the time of initial learning rather than at retrieval.

Two main accounts have been proposed to explain this effect. The selective rehearsal account suggests that TBR items receive elaborative encoding, whereas rehearsal is discontinued for TBF items, leading to weaker memory traces (Basden et al., 2000; Jing et al., 2019). In contrast, inhibition-based accounts propose that forget cues trigger active control processes that suppress or downregulate encoding or subsequent accessibility of TBF representations (Anderson & Hulbert, 2021; Fawcett et al., 2016; Fawcett & Taylor, 2008). More recent work suggests that these mechanisms may operate alongside broader encoding and contextual processes (Sahakyan, 2024). For example, evidence indicates that successful forgetting may involve the separation or “unbinding” of item and contextual information, reducing the likelihood of later retrieval (Chiu et al., 2021). Similarly, encoding strength has been identified as a critical determinant of DF effects, with stronger or more distinctive representations being less susceptible to intentional forgetting (Hauswald et al., 2011; Spear et al., 2024). Furthermore, memories perceived as critical for immediate cognitive tasks are often retained despite attempts to forget (Festini & Reuter-Lorenz, 2017), while emotional stimuli often resist such instructions (Bailey & Chapman, 2012; Hall et al., 2021; Nowicka et al., 2011). Taken together these accounts suggest that directed forgetting is not a unitary process but depends on the interaction between control mechanisms and the strength and structure of the encoded memory trace.

In this context, expectation violation may represent a critical boundary condition for memory control. If unexpected events enhance encoding strength through prediction-error mechanisms, they may reduce the effectiveness of directed forgetting, as the resulting memory traces may be less dependent on continued rehearsal or more resistant to suppression. Although directed forgetting has been shown to influence both recollection- and familiarity-based recognition (Festini & Reuter-Lorenz, 2014; Gao et al., 2019), the underlying mechanisms are thought to operate at encoding, affecting the strength and structure of memory representations (e.g., Anderson & Hulbert, 2021; Gao et al., 2019; Macleod, 1999). Because recollection is more sensitive to variations in encoding strength and associative processing, we predicted that the effects of expectation violation on DF would be more pronounced for recollection than familiarity. The present study tests whether prediction error reduces the effectiveness of directed forgetting, and whether this effect is modulated by emotional valence.

Using an item-method DF paradigm with both emotional and neutral stimuli, we investigated whether the mnemonic advantage of unexpected items persists even when participants are instructed to forget them. Based on predictive-processing accounts, we hypothesised that expectation violation would enhance encoding strength and increase the likelihood of recollection relative to familiarity. Given that DF is most effective for weakly encoded representations, we further predicted that this enhancement would reduce the effectiveness of DF. Specifically, (1) unexpected items would be remembered better than expected items, particularly through recollection; (2) DF effects would be attenuated for unexpected relative to expected items, reflecting reduced susceptibility to forgetting; and (3) emotional valence would modulate these effects, such that conditions associated with stronger encoding (e.g., negative or arousing stimuli) may further influence the extent to which expectation violation reduces DF. (4)Finally, based on prior evidence that unexpected events are more likely to elicit recollection-based recognition, we examined whether the predicted attenuation of directed forgetting for unexpected items was expressed primarily in recollection, familiarity, or both. Establishing such an effect would identify expectation violation as an important determinant of memory control, complementing evidence that emotional salience constrains intentional forgetting. More broadly, clarifying how unexpected information reduces susceptibility to suppression may inform approaches to enhance durable learning, for example by leveraging expectation-violating elements to strengthen long-term retention.

## Materials and Methods

### Participants

A total of 117 participants (43 female) consented to take part in the study via Prolific and received monetary compensation. Seventeen were excluded for performing at or below chance on the recognition memory task, indicating insufficient engagement. A further 22 were excluded for scoring below 60% accuracy on the rule-learning task, suggesting limited acquisition of the contingencies and thus inadequate expectation formation, which was central to the paradigm. These cut-offs were defined a priori, and inclusion of excluded participants did not change the overall direction of the critical effects. The final sample comprised 78 participants (43 male; mean age: 28.91; SD = 4.69). Eligibility criteria required participants to be 18–35 years old, with no history of neurological or psychiatric conditions, and with normal or corrected-to-normal vision. Ethical approval was obtained from the University of Manchester ethics committee. All participants were recruited through Prolific (https://www.prolific.com) and completed the experiment online using Gorilla (www.gorilla.sc). A sensitivity power analysis was conducted to estimate the smallest effect size detectable with the present sample size (*N* = 78). Assuming a conventional alpha level of 0.05 and 80% power, the study was sufficiently powered to detect small-to-medium effects, which are typical of prior research on expectation violation and DF. In addition, the sample size is comparable to previous studies using similar paradigms (e.g., Kafkas, 2021; Mukhtar & Kafkas, 2026; Spear et al., 2024).

### Stimuli

Emotional and neutral images were drawn from the International Affective Picture System (IAPS; Lang et al., 2008). Selection was based on two dimensions: (1) valence (positive, negative, neutral) and (2) arousal (calm to excited). Negative (M = 5.40, SD = 0.70) and positive stimuli (M = 4.78, SD = 0.80) were more arousing than neutral stimuli (neutral: M = 3.30, SD = 0.70; *t*(186) = 21.48, *p* <.001 and *t*(186) = 13.32, *p* <.001, respectively), while negative stimuli were more arousing than positive stimuli (t(186) = 6.17, *p* <.001. In addition, six abstract symbols from Kafkas (2021) were used.

### Design and Procedure

**Fig. 1:**
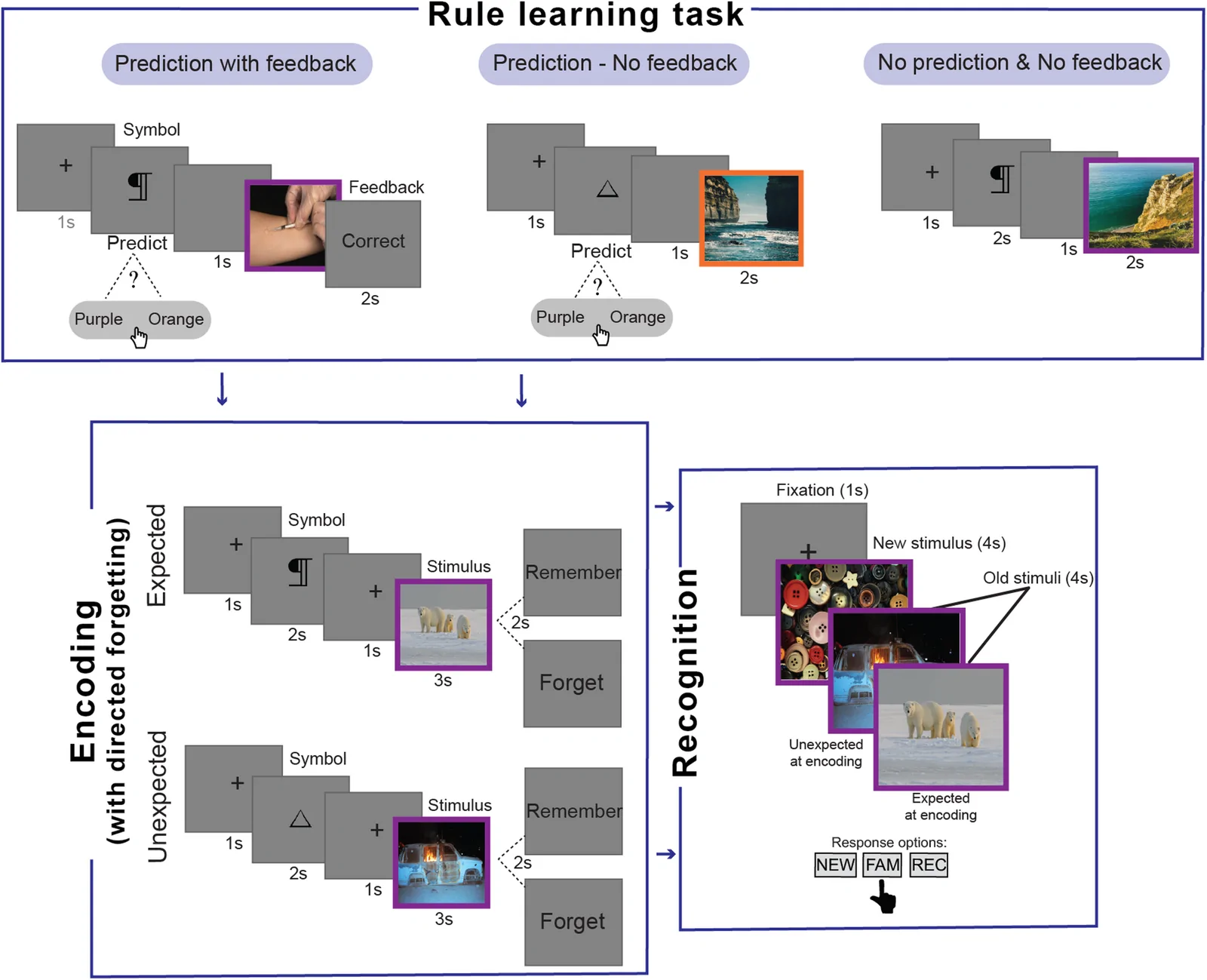
Experimental design. The figure illustrates the rule learning, encoding, and recognition memory tasks, with an item-method directed forgetting procedure implemented at encoding. Neutral, negative, and positive stimuli were used throughout. The rule learning task established contingency associations between cue symbols and stimulus frames. During encoding, a new set of stimuli appeared following the same cues from the rule learning phase. 60% of trials followed the learned rule (expected stimuli), and 40% violated it (unexpected stimuli). Examples of expected and unexpected event sequences are shown. Each stimulus was followed by a ‘remember’ or ‘forget’ instruction; participants were told they would later be tested only on the ‘remember’ items. In the recognition memory task, previously encoded (expected/unexpected × remember/forget) stimuli were presented intermixed with new foils. Participants classified each item as new (NEW), familiar (FAM), or recollected (REC). The images shown are for illustrative purposes only; they are copyright-open examples and not the actual IAPS stimuli used in the experiments.

The experiments were based on previous work in which expectation was manipulated at encoding through contingency learning (Kafkas 2021; Kafkas & Montaldi 2018a), here extended to emotional stimuli (as in Mukhtar & Kafkas, 2026). Expectation was established during a rule-learning task and manipulated at encoding (see Fig. 1). All participants completed three phases in sequence: rule learning, encoding with directed forgetting, and recognition memory. The study was administered online, and participants were required to use a computer (PC or laptop) with a mouse and keyboard. They were instructed to complete the experiment in a quiet, distraction-free environment and in a single sitting, which was verified through completion times, but no participants were excluded on this basis alone.

#### Rule Learning Task

Participants learned to associate six abstract symbols with either a purple- or an orange-framed stimulus. In the first cycle, 72 unique stimuli (36 symbol–stimulus pairs, six repetitions per symbol) were shown. Participants predicted the colour of the frame (purple or orange) that would follow each symbol. In the first two cycles, this prediction phase was self-paced and the symbol remained on screen until the participant made a response. After each prediction, a picture appeared for 3 s with the corresponding frame, followed by feedback indicating whether the prediction was correct (see Fig. 1). Each cue symbol was repeated nine times across the session, paired with different emotional (negative, positive) or neutral stimuli, evenly distributed across colours. Three symbols were consistently followed by purple-framed stimuli and three by orange-framed stimuli, with symbol–colour assignments randomised per participant. In the second cycle, 18 symbol–stimulus pairs were presented with new pictures, predictions were again required, but no feedback was given. In the third cycle, the same 18 trials were shown without predictions or feedback. In this cycle no overt responses were required, and each symbol was presented for 2 s before the stimulus appeared. Participants were instructed to continue attending to the symbol–stimulus sequences. This final cycle was included to facilitate a more natural transition to the encoding phase, during which no response related to each symbol was required, and to ensure that learned expectations were maintained under passive viewing conditions. This procedure follows previous work using the same paradigm (Kafkas 2021; Kafkas & Montaldi 2018a). Learning was assessed through accuracy and reaction time (RT) across cycles; high accuracy and reduced RTs confirmed successful contingency learning before encoding (see Results).

#### Encoding Task

Participants were informed that the same symbols would reappear, paired with new framed stimuli. Expectation was manipulated by either maintaining or violating the learned symbol–colour contingencies. Specifically, 60% of trials followed the learned rules (expected) and 40% violated them (unexpected). The proportion of expected (60%) and unexpected (40%) trials was selected to balance the need to elicit prediction errors with maintaining a stable predictive structure. Such probabilistic contingencies are standard in prediction-error paradigms, where violations are defined relative to an established regularity. Previous work using this paradigm has shown that this ratio produces robust and sustained expectancy effects across encoding (Kafkas 2021; Kafkas & Montaldi 2018a), while higher proportions of unexpected trials can reduce effective prediction error (Kafkas et al., 2025). A total of 120 stimuli were presented (24 expected and 16 unexpected per emotion). After each stimulus, participants received a “remember” or “forget” instruction (with a 50:50 ratio). They were told only “remember” items would later be tested. Each trial comprised: fixation cross (1 s), cue symbol (2 s), blank screen (1 s), stimulus (2 s), and remember/forgetting instruction (2 s). Participants were informed that, in the encoding task, the same symbols would appear as in the rule-learning phase, but that no prediction responses would be required. Instead, they were instructed to focus on the pictures, which constituted the target stimuli for the memory task, and to follow the instructions to either remember or forget each picture. The assignment of images to expected and unexpected conditions was randomised and counterbalanced across participants, such that each image appeared equally often in each condition across the sample. Furthermore, emotional valence (negative, positive, neutral) was balanced across expected and unexpected conditions.

#### Recognition Memory Task

Following a 7-min arithmetic filler task, participants completed the recognition memory test. Beforehand, they received training to distinguish familiarity from recollection. Familiarity was defined as recognising a picture as previously seen without retrieving any specific detail from its earlier presentation. For example, a participant might feel that a picture was old but be unable to recall anything else about the study episode. Recollection, by contrast, required retrieval of additional details from encoding (Kafkas et al., 2017; Montaldi & Kafkas, 2024; Montaldi & Mayes, 2010). For example, a participant might remember not only that the picture had been presented before, but also a specific thought or association they had generated at the time, such as finding the image unusual or relating it to a personal memory. During the task, participants had 4 s to identify each stimulus as new, familiar, or recollected. The expected/unexpected manipulation applied only to studied items during encoding, as it was defined by the learned relationship between the cue symbol and frame colour. New items presented at test were not associated with any prior cue-based expectancy state and therefore were not classifiable as expected or unexpected. Pictures were presented within the same coloured frame in which they had appeared at encoding, to preserve continuity across phases of the task. Frame colour was therefore consistent with encoding but was not manipulated at test. New stimuli were assigned either a purple or an orange frame at random during the recognition test, ensuring that frame colour was not diagnostic of old/new status and could not guide memory judgments. Participants were instructed to base their responses on whether they had previously seen the picture itself. All stimuli presented at encoding (120 trials) were included along with 90 new (non-studied) stimuli making a total of 210 trials.

### Data Analyses

Recognition performance was computed as corrected recognition (hit rate minus false alarm rate) within each condition. Sensitivity (d’) was also computed as *z*(hit rate) – *z*(FA), but as it produced comparable patterns, these results are not reported separately. False alarm rates were computed separately for each emotional category (neutral, negative, positive) and for each response type (recollection, familiarity), such that each condition had its own corresponding false alarm rate. Because participants made a single three-way recognition judgment, familiarity and recollection responses were mutually exclusive. For old items, the total hit rate was therefore equal to the sum of familiarity hits and recollection hits, with the remaining responses classified as misses. For new items, false familiarity and false recollection responses were likewise mutually exclusive, with the remaining responses classified as correct rejections. Participant-by-condition cells with zero responses in a given category were retained as zero values when calculating proportions. Corrected recognition scores were then calculated by subtracting the appropriate false alarm rate from the hit rate within each condition.

In the rule-learning task, accuracy (correct/incorrect) was analysed using a logistic mixed-effects model with symbol repetition (1–6), image valence (negative, positive, neutral), and symbol type (six symbols) as fixed factors and participants as a random intercept. Similarly, RTs in the same task were analysed using a linear mixed-effects model with the same fixed-effects structure and participants as a random intercept. Memory performance was analysed using linear mixed-effects models with expectation (expected vs. unexpected), DF instructions (forget, remember), and emotion (negative, positive, neutral) as fixed effects, and participants as a random intercept. Analyses were run separately for familiarity and recollection performance, with an additional complementary analysis conducted on overall corrected recognition performance collapsed across response types. Reaction times were analysed separately for familiarity and recollection responses using the same modelling approach. As memory performance is calculated at the condition level, item-level variability was not modelled in the corresponding mixed-effects analyses. The mixed effects models were run in R (lme4; Bates et al., 2015) and Type III Wald F tests with Satterthwaite’s approximation of degrees of freedom (Kuznetsova et al., 2017; Satterthwaite, 1946) are reported. As reported above, all mixed-effects models included random intercepts for participants. We additionally explored random slopes for the main effects, but these were not retained because the corresponding models either failed to converge or did not improve fit according to likelihood-ratio tests. Final models therefore used a random-intercepts-only structure. Effect size, in the mixed-effects models, is reported using marginal and conditional R², reflecting the variance explained by the fixed effects alone and by the full model (fixed plus random effects), respectively. Significant effects were followed up with Bonferroni-corrected pairwise comparisons. A significance threshold of *p* <.05 was applied throughout, while Greenhouse-Geisser corrections were applied in case of sphericity violations (*p*-values noted as *p*_GG_).

## Results

### Rule Learning

**Fig. 2:**
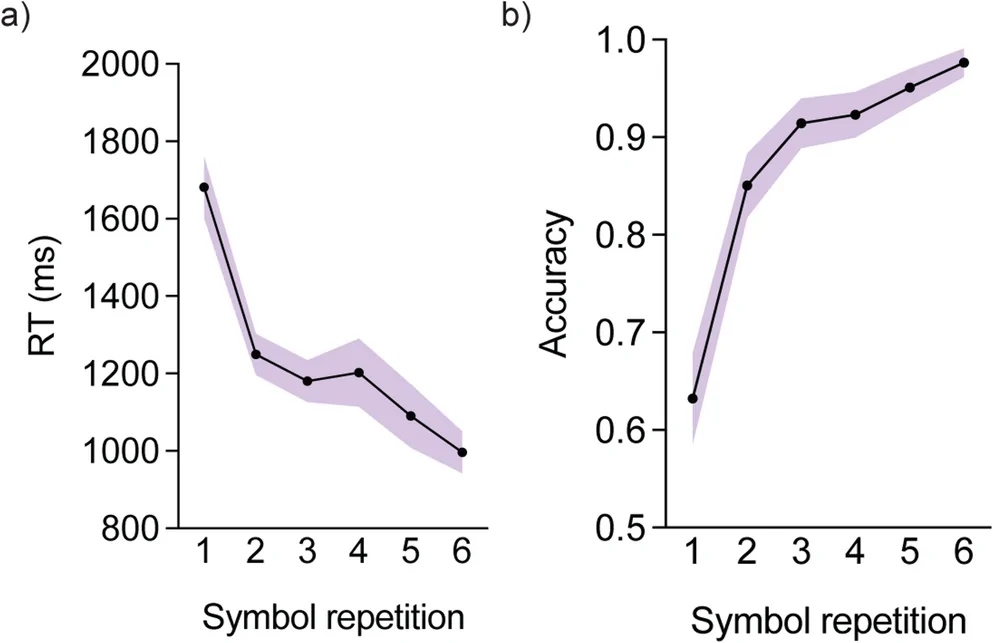
Reaction times (RTs; **a**) and prediction accuracy (**b**) in the rule learning task across cue symbol repetitions. Significant decreases in RTs and increases in accuracy indicate successful learning of the contingency associations, reflecting the development of clear expectations for symbol–stimulus sequences

Participants demonstrated strong rule learning, with mean accuracy significantly above chance (*M* = 0.74, *SD* = 0.05; one-sample *t*-test against 0.50: *t*(77) = 40.14, *p* <.001, *d* = 0.38). Prediction accuracy increased and response times (RTs) decreased significantly across symbol–stimulus repetitions as shown by the significant main effect of symbol repetition (RTs: *F*(5, 2730) = 12.01, *p* <.001, R^2^_marginal_ = 0.03, R^2^_conditional_ = 0.10; Accuracy: *χ*^*2*^(5) = 153.82.17, *p* <.001;, R^2^_marginal_ = 0.86, R^2^_conditional_ = 0.87;see Fig. 2). No significant effects of valence (RTs: *F* < 1; Accuracy: *χ*^*2*^ < 1) or symbol type (RTs: *F* < 1; Accuracy: *χ*^*2*^(3) = 3.18, *p* =.36) were observed for either measure, suggesting comparable learning trajectories across emotional conditions and symbol types. These findings indicate that the task successfully established expectations regarding the frame of upcoming stimuli.

### Effect of Directed Forgetting on Subsequent Memory

#### Performance

The mean proportions across the different response outcomes (hits, false alarms, misses and correct rejections) across expectation and DF instructions are summarised in Table 1.

**Table 1:** Mean proportions (and standard deviations) of recognition memory outcomes as a function of cue type, emotion, and expectation

		Neutral		Negative		Positive	
	Cue	Expected	Unexpected	Expected	Unexpected	Expected	Unexpected
Hit_FAM	Remember	0.31 (0.24)	0.29(0.26)	0.32 (0.21)	0.30 (0.22)	0.28 (0.19)	0.29 (0.18)
	Forget	0.34 (0.23)	0.32 (0.26)	0.36 (0.22)	0.33 (0.18)	0.37 (0.20)	0.33 (0.22)
Hit_REC	Remember	0.29 (0.24)	0.39 (0.27)	0.35 (0.22)	0.45 (0.26)	0.41 (0.21)	0.36 (0.23)
	Forget	0.21 (0.20)	0.36 (0.26)	0.25 (0.20)	0.38 (0.19)	0.23 (0.20)	0.25 (0.22)
M	Remember	0.40 (0.23)	0.32 (0.23)	0.33 (0.17)	0.25 (0.18)	0.31 (0.19)	0.35 (0.21)
	Forget	0.45 (0.22)	0.32 (0.22)	0.39 (0.20)	0.29 (0.14)	0.40 (0.20)	0.42 (0.25)
CR		0.82 (0.20)	0.82 (0.20)	0.71 (0.22)	0.71 (0.22)	0.79 (0.22)	0.79 (0.22)
FA_FAM		0.15 (0.12)	0.15 (0.12)	0.23 (0.18)	0.23 (0.18)	0.19 (0.16)	0.19 (0.16)
FA_REC		0.03 (0.04)	0.03 (0.04)	0.07 (0.07)	0.07 (0.07)	0.03 (0.08)	0.03 (0.08)

Values represent mean proportions with standard deviations (SD) in parentheses. Hit_FAM = hits associated with familiarity responses; Hit_REC = hits associated with recollection responses; CR = correct rejections; M = misses; FA_FAM = false alarms associated with familiarity responses; FA_REC = false alarms associated with recollection responses

**Fig. 3:**
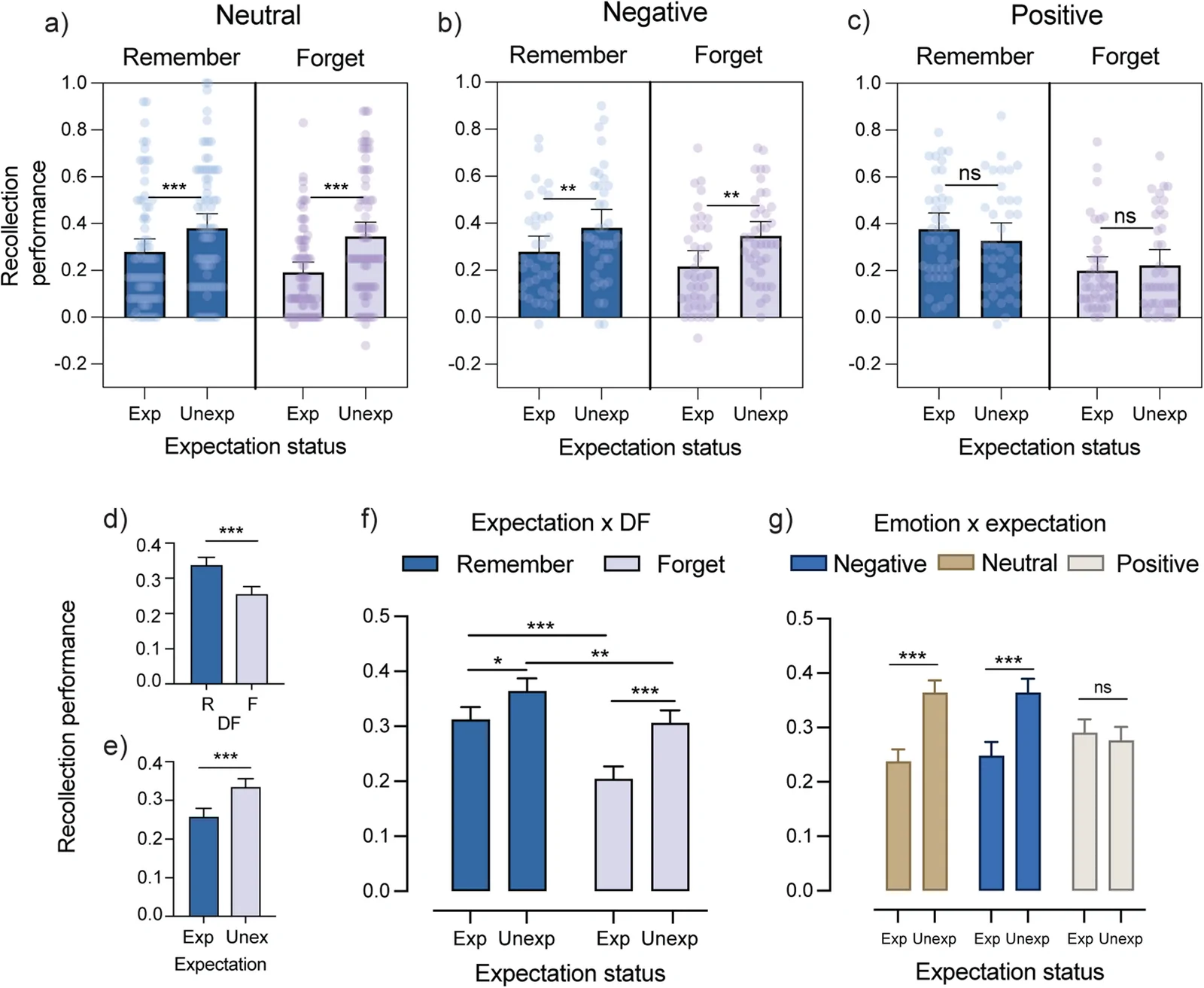
Recollection performance across emotion, expectation, and directed forgetting (DF) conditions. Panels (**a**–**c**) show recollection performance for neutral, negative, and positive stimuli, respectively, as a function of expectation status (expected, unexpected) and DF instruction (remember, forget). Panels (**d**–**e**) present the main effects of DF and expectation, respectively, indicating higher recollection for ‘remember’ than ‘forget’ items (**d**) and for unexpected than expected stimuli (**e**). Panel (**f**) illustrates the expectation × DF interaction, with unexpected–remember items showing the highest recollection and a sustained effect of unexpectedness even for ‘forget’ trials. Panel (**g**) shows the emotion × expectation interaction, with a stronger expectation effect for negative and neutral stimuli than for positive stimuli. Error bars represent 95% confidence intervals

The linear mixed-effect model analysis on recollection performance (Hits – FAs; R^2^_marginal_ = 0.08, R^2^_conditional_ = 0.65) revealed a significant effect of DF instructions (*F*(1, 544) = 50.59, *p* <.001, *β* = 0.08) with higher performance for “remember” than “forget” trials (*M* = 0.34, *SE* = 0.02 vs. *M* = 0.25, *SE* = 0.02, respectively). The main effect of emotion was not significant (*F*(2, 544) = 1.23, *p* =.29), suggesting that recollection performance did not differ reliably across the three emotional conditions. The main effect of expectation was significant (*F*(1, 544) = 43.20, *p* <.001, *β* = 0.08), reflecting greater recollection for unexpected than expected stimuli (*M* = 0.33, *SE* = 0.02 vs. *M* = 0.26, *SE* = 0.02, respectively). A significant emotion × expectation interaction (*F*(2, 544) = 14.68, *p* <.001; Fig. 3) indicated that the expectation effect differed by emotion: unexpected stimuli were remembered better than expected stimuli when negative (*t*(544) = − 5.21, *p*_bonf_ < 0.001, 95% CI [−0.12, −0.07]), or neutral (*t*(544) = − 8.20, *p*_bonf_ < 0.001, 95% CI [−0.16, −0.09]), but not when positive (*t* < 1). Importantly, the expectation × DF instruction interaction was also significant, (*F*(1, 544) = 4.72, *p* =.03) indicating differential DF effect for expected and unexpected stimuli. Specifically, “forget” instruction reduced recollection significantly more for expected than unexpected stimuli (*t*(544) = − 6.20, *p*_bonf_ < 0.001, 95% CI [−0.13, −0.07]), whereas “remember” instructions enhanced recollection for unexpected stimuli more than expected (*t*(544) = − 3.10, *p*_bonf_ = 0.012, 95% CI [−0.08, −0.02]; Fig. 3). The three-way interaction was not significant (*F* < 1), suggesting that the effect of directed forgetting by expectation did not differ reliably across emotional conditions.

**Fig. 4:**
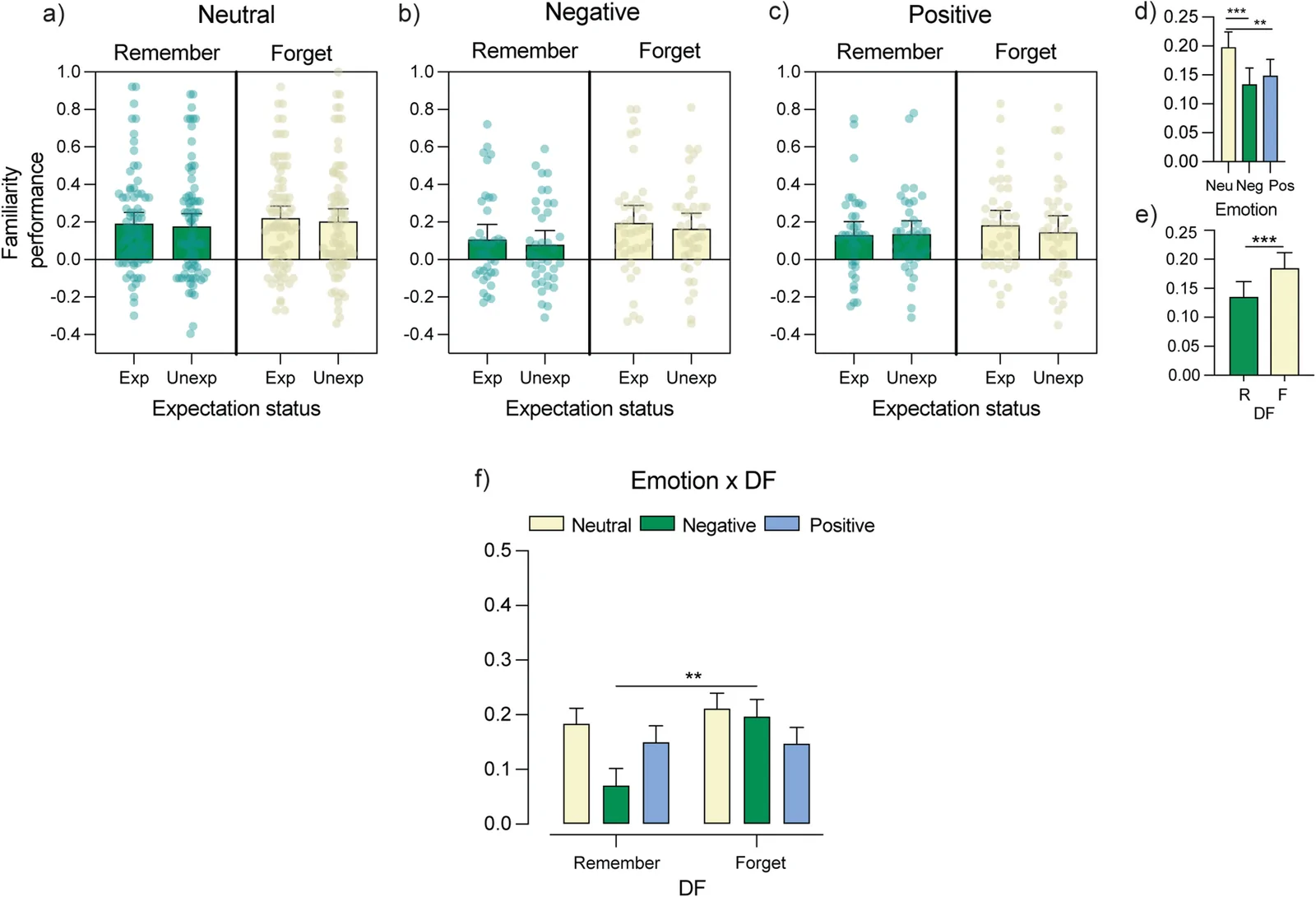
Familiarity performance across emotion, expectation, and directed forgetting (DF) conditions. Panels (**a**–**c**) show familiarity performance for neutral, negative, and positive stimuli, respectively, as a function of expectation status (expected, unexpected) and DF instruction (remember, forget). Panel (**d**) depicts the main effect of emotion, showing higher familiarity for neutral than negative or positive stimuli. Panel (**e**) presents the main effect of DF, indicating higher familiarity for ‘forget’ than ‘remember’ items. Panel (**f**) illustrates the emotion × DF interaction, with greater familiarity for neutral than positive stimuli on ‘forget’ trials. Error bars represent 95% confidence intervals

The mixed model analysis on familiarity performance (R^2^_marginal_ = 0.03, R^2^_conditional_ = 0.71) revealed a significant main effect of DF (*F*(1, 544) = 16.78, *p* <.001, *β* = 0.05), with higher familiarity performance for “forget” than “remember” trials (M = 0.18, SE = 0.03 vs. M = 0.13, SE = 0.03). The main effect of expectation was not significant (*F*(1, 544) = 2.75, *p* =.10), suggesting that familiarity did not differ reliably between expected and unexpected stimuli. In contrast, the main effect of emotion was significant (*F*(2, 544) = 12.20, *p* <.001), with greater overall familiarity for neutral stimuli compared to both negative (*t*(544) = − 4.47, *p*_bonf_ < 0.001, 95% CI [−0.09, −0.04]) and positive stimuli (*t*(544) = 3.46, *p*_bonf_ = 0.002, 95% CI [0.02, 0.08]). The emotion × DF instruction interaction was also significant (*F*(2, 567) = 5.08, *p* =.007), reflecting a reduction in familiarity for “remember” compared with “forget” trials in the negative (*t*(576) = 4.48, *p* <.001, 95% CI [0.07, 0.18), but not in the positive or neutral conditions (*t* < 1 and *t*(544) = 1.67, *p*_bonf_ = 0.55, respectively; see Fig. 4).

To complement the process-specific analyses, we conducted an additional analysis of overall corrected recognition performance (hits minus false alarms), collapsing across recollection and familiarity responses (R^2^_marginal_ = 0.05, R^2^_conditional_ = 0.74). This analysis revealed significant main effects of DF instruction (*F*(1, 544) = 7.26, *p* =.007, *β* = 0.03; TBR > TBF), expectation (*F*(1, 544) = 21.75, *p* <.001, *β* = 0.06; unexpected > expected), and emotion (*F*(2, 544) = 15.28, *p* <.001, *β* = 0.06; neutral > negative & neutral > positive). It also showed a significant emotion × expectation interaction (*F*(2, 544) = 13.01, *p* <.001), with greater increase in recognition memory performance for unexpected than expected stimuli in the case of negative and neutral items (*t*(544) = −3.76, *p =*.002 and *t*(544) = −6.91, *p <*.001, respectively) but not for positive items (*t*(544) = 1.28, *p =*.77), whereas the DF instruction × expectation interaction was not significant (*F*(1, 544) = 2.01, *p* =.156) and the three-way interaction remained non-significant (*F* < 1). Thus, directed forgetting and expectation both influenced overall corrected recognition memory. Although familiarity-based responding was higher for TBF than TBR items, overall corrected recognition was higher for TBR than TBF items because the TBR advantage in recollection outweighed the reverse pattern observed for familiarity. Importantly, however, the attenuation of directed forgetting for unexpected items was not reliable in overall corrected recognition but was most clearly evident in recollection-based memory.

#### Response Times

**Fig. 5:**
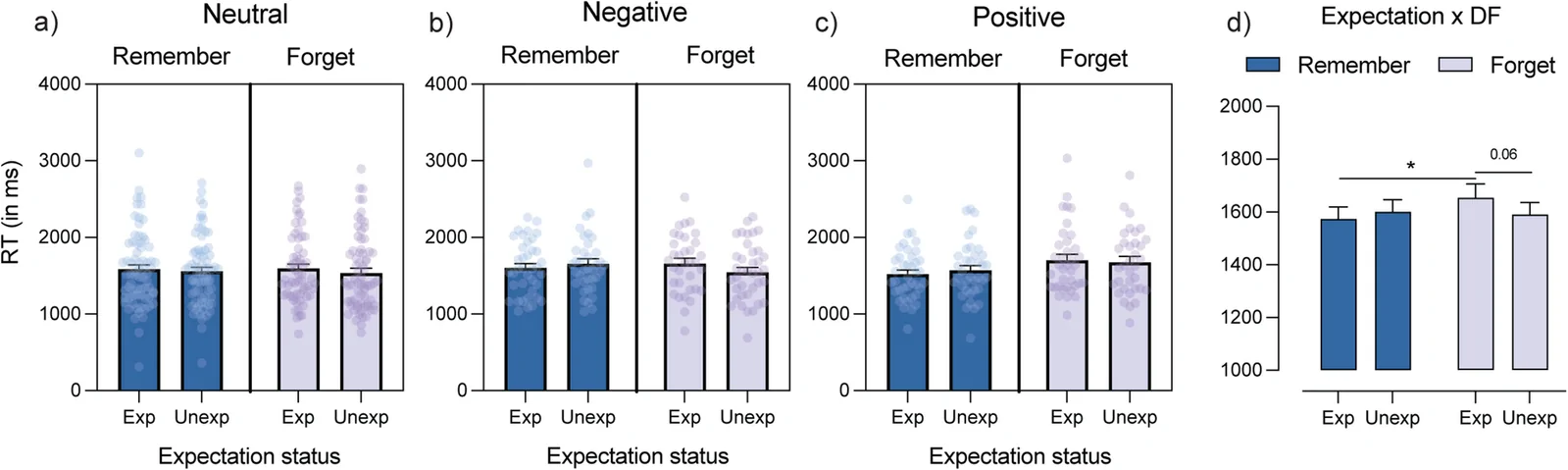
Reaction times (RTs) during encoding for subsequently recollected items, across emotion, expectation, and directed forgetting (DF) conditions. Panels (**a**–**c**) show RTs for neutral, negative, and positive stimuli, respectively, as a function of expectation status (expected, unexpected) and DF instruction (remember, forget). Panel (**d**) shows the pattern of RTs across expectation and DF conditions. Although the omnibus expectation × DF interaction did not reach significance (*p* =.055), follow-up comparisons indicated longer RTs for expected stimuli on ‘forget’ than on ‘remember’ trials (*p* =.018), and a trend towards shorter RTs for unexpected than expected stimuli on ‘forget’ trials (*p* =.06). The asterisk denotes the significant pairwise comparison, not the omnibus interaction. Error bars represent 95% confidence intervals

The mixed-effects model analysis of RTs for recollections (R^2^_marginal_ = 0.01, R^2^_conditional_ = 0.63) revealed no significant main effect of DF (*F*(1, 489) = 2.06, *p* =.15), emotion (*F*(2, 488) = 1.08, *p* =.34), or expectation (*F* < 1; see Fig. 5). None of the interactions reached significance (all *p*s > 0.05), although there was a strong trend for the DF × expectation interaction (*F*(1, 488) = 3.70, *p* =.055; Fig. 5d). Follow-up comparisons revealed a trend towards shorter RTs for unexpected compared to expected *forget* trials (*t*(488) = 1.88, *p*_*bonf*_ = 0.06, 95% CI [−3.69, 131.60]), and significantly shorter RTs for *remember* than *forget* trials in the expected condition (*t*(488) = 2.37, *p*_*bonf*_ = 0.018. 95% CI [12.85, 146.10]). No difference was observed between *remember* and *forget* trials in the unexpected condition (*t* < 1).

**Fig. 6:**
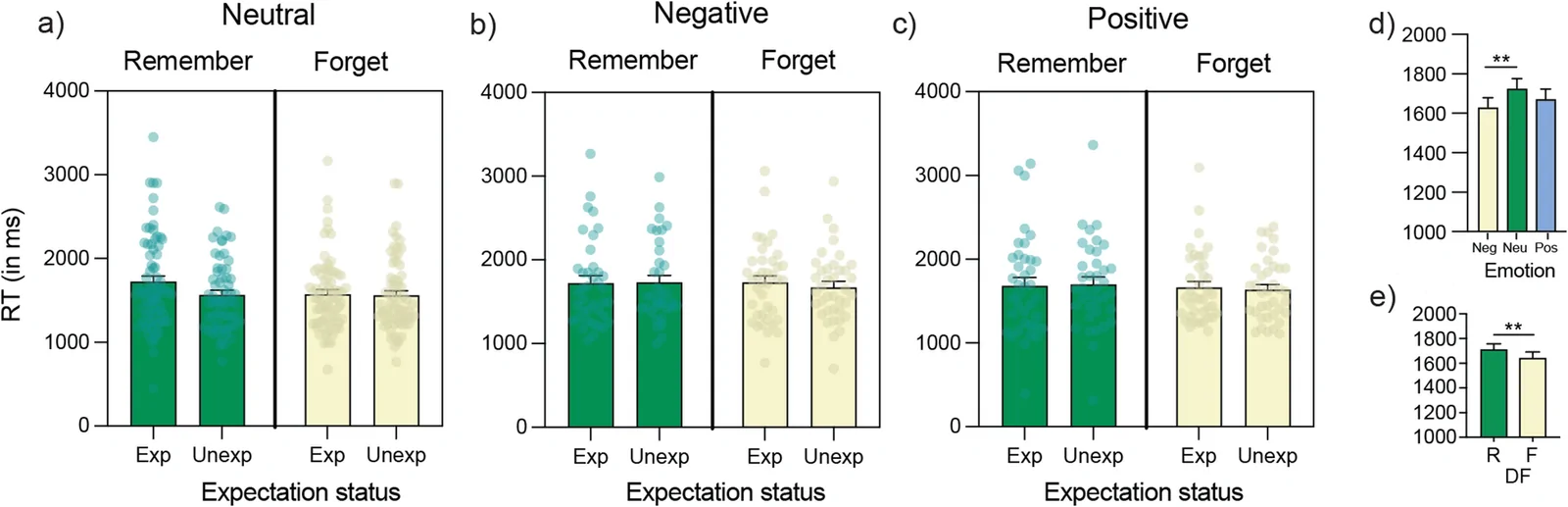
Reaction times (RTs) during encoding for subsequently familiar items across emotion, expectation, and directed forgetting (DF) conditions. Panels (**a**–**c**) show RTs for neutral, negative, and positive stimuli, respectively, as a function of expectation status (expected, unexpected) and DF instruction (remember, forget). Panel (**d**) presents the main effect of emotion, with longer RTs for negative than neutral stimuli. Panel (**e**) presents the main effect of DF, showing longer RTs for ‘remember’ than for ‘forget’ trials. Error bars represent 95% confidence intervals

The RT analysis for familiarity responses (R^2^_marginal_ = 0.02, R^2^_conditional_ = 0.64) revealed a significant main effects of DF (*F*(1, 497) = 7.00, *p* =.008) and emotion (*F*(2, 496) = 5.06, *p* =.007; see Fig. 6), with shorter RTs for ‘forget’ than ‘remember’ trials and in the case of neutral versus negative trials (*t*(497) = 3.17, *p* =.005, 95% CI [35.30, 153.60]). The main effect of expectation was not significant (*F* < 1), nor were any of the interactions in this analysis (all *p*s > 0.05).

## Discussion

The present study examined whether expectation violations modulate the effectiveness of directed forgetting (DF). We reasoned that deliberate attempts to forget may be less effective when stimuli are unexpected, as such events may be less susceptible to the mechanisms ordinarily proposed to support DF, including reduced rehearsal in selective-rehearsal accounts (Macleod, 1999) and active suppression in inhibition-based accounts (Fawcett et al., 2016; Fawcett & Taylor, 2008). It was predicted that unexpected stimuli would be better remembered through recollection (Kafkas 2021; Kafkas & Montaldi 2018a; Mukhtar & Kafkas 2026; Kafkas et al. 2025) and that this memory advantage would persist under forget instructions. Consistent with these predictions, unexpected stimuli were remembered better than expected ones—particularly in terms of recollection—and proved more resistant to DF. Although ‘forget’ instructions reduced recollection overall, this effect was markedly greater for expected than for unexpected stimuli. By contrast, familiarity was higher for TBF than TBR items and was not reliably modulated by expectation, consistent with previous evidence linking familiarity to more automatic encoding processes (Migo et al., 2012; Montaldi & Kafkas, 2024). We further hypothesised that emotional stimuli would enhance the recollection advantage for unexpected events. This was not supported in a general sense, as neutral stimuli showed a comparable expectation effect to negative stimuli. However, the observed pattern was partly consistent with the more specific expectation that negative stimuli would show a greater expectation advantage than positive stimuli. Finally, the predicted selective resistance to forgetting for unexpected negative stimuli was not observed; instead, resistance to DF for unexpected stimuli occurred across all emotional categories.

### Expectation and Emotion

Recent evidence suggests that mnemonic advantage of expectation-violating events is not uniform but depends on dispositional traits (Kafkas et al., 2025) and more importantly, for the present study, on emotional valence, arousal, and context (Mukhtar & Kafkas, 2026). Specifically, Mukhtar and Kafkas (2026) recently showed that unexpected stimuli enhanced recollection for negative and neutral items but not for positive ones (in mixed lists) and that list context modulated this effect, as the effect vanished in pure emotional lists. These findings align closely with the present results, where unexpected stimuli boosted recollection most strongly for negative and neutral stimuli. Such convergence implies that the prediction-error encoding benefit is modulated by both emotional valence and contextual factors.

One possible interpretation is that negative stimuli may attract greater attention and elicit deeper encoding, than positive stimuli, particularly when they are unexpected (Keltner & Gross, 1999; Kensinger et al., 2007; Mather & Sutherland, 2011; Plutchik et al., 2003). Positive unexpected events, by contrast, may not generate the same degree of attentional capture (Kensinger et al., 2007) or may engage different encoding pathways (Williams et al., 2022), for example because of differences in motivational orientation such as approach versus avoidance. However, the present pattern does not support a simple arousal-based account. In particular, although positive stimuli were lower in arousal than negative stimuli, neutral stimuli were lower in arousal than both emotional categories yet showed an expectation effect more similar to negative than to positive items. Thus, while arousal may have contributed to the difference between negative and positive stimuli (Zhao & Zhang, 2025), it cannot fully explain the overall pattern.

This more cautious interpretation is also consistent with Mukhtar and Kafkas (2025), who found that arousal-matching shifted the expectation benefit to positive and neutral stimuli, indicating that the interaction between expectation and emotion is context-sensitive rather than fixed. More broadly, emotional memory effects are not reducible to a single valence or arousal dimension, as positive and negative episodic memories differ in complex ways (Williams et al., 2022), and the relative distinctiveness or organisation of neutral and emotional items may also shape memory performance (Talmi et al., 2007; Talmi & Moscovitch, 2004). The present findings therefore suggest that the interaction between expectation and emotion depends on additional stimulus and contextual factors beyond arousal alone.

This interpretation fits within predictive-coding accounts of memory formation, in which violations of expectation elicit dopaminergic and hippocampal engagement to promote new learning (Frank & Kafkas 2021; Kafkas & Montaldi 2018b). When emotional arousal is also high, these mechanisms may interact with amygdala–hippocampal circuits to strengthen consolidation (LaBar & Cabeza, 2006; Schott et al., 2011). Our data suggest that emotion and expectation may operate jointly, with each amplifying or constraining the other’s effect on recollection. At the same time, the absence of an expectation effect for positive stimuli indicates that only some combinations of expectancy violation and emotional properties produce this recollective advantage, and that the relevant boundary conditions remain to be specified.

Finally, it is important to note that expectation in the present paradigm is assumed to operate implicitly. Although participants were instructed to focus on the images and directed forgetting cues during encoding, expectations established during the rule-learning phase were likely to be generated automatically at encoding, such that violations elicited prediction-error signals even in the absence of explicit attention to the predictive cues. This interpretation is consistent with previous work using this paradigm (Kafkas, 2021; Kafkas et al., 2025; Mukhtar & Kafkas, 2026), demonstrating that expectation-related effects on memory emerge even when cues are task-irrelevant. Within this framework, expectation violation functions as an implicit signal that enhances encoding strength, thereby influencing subsequent memory performance.

### Expectation, Emotion and Directed Forgetting

The key novel contribution of this study lies in demonstrating that expectation status alters the effectiveness of directed forgetting. Specifically, the forget instruction reduced recollection to a greater extent for expected than for unexpected items. Furthermore, the overall effect of unexpectedness (i.e., difference in recollection between expected and unexpected stimuli) was greater in the case of ‘forget’ trials. The present findings indicate that expectation violation reduces the effectiveness of DF, particularly for recollection-based memory. This pattern can be interpreted within both major accounts of item-method DF. According to selective rehearsal accounts, forget cues lead to the discontinuation of elaborative processing, resulting in weaker memory traces for TBF items (Macleod, 1999). However, if unexpected events trigger enhanced encoding through prediction-error mechanisms, these items may already possess sufficiently strong and distinctive representations that are less dependent on continued rehearsal, thereby reducing the impact of the forget instruction. From an inhibition-based perspective, DF is thought to involve active suppression of memory representations (Anderson & Hulbert, 2021). In this case, the enhanced encoding associated with expectation violation may produce memory traces that are more resistant to suppression, for example due to stronger hippocampal binding or increased distinctiveness. Importantly, although these accounts differ in mechanism, both predict reduced forgetting for strongly encoded items. The present findings therefore suggest that prediction error acts as a boundary condition on DF by strengthening encoding beyond the level at which forgetting processes operate effectively.

Finally, unexpected events were not just better remembered but were retrieved more fluently when recollected as revealed by the RT effects. Importantly, shorter response latencies were found for unexpected compared with expected ‘forget’ trials, while longer RTs were found for expected stimuli on ‘forget’ than ‘remember’ trials (Fig. 5d). This pattern suggests that violations of expectation not only enhanced recollection accuracy but also facilitated retrieval efficiency when participants recognised previously unexpected items, even those instructed to be forgotten. The longer RTs for expected ‘forget’ trials may reflect increased decisional conflict, as participants encountered stimuli that fit well-learned contingencies yet lacked strong contextual reinstatement cues due to earlier suppression attempts. Unexpected items, by contrast, might have formed distinctive memory representations during encoding (Kafkas & Montaldi, 2018a), enabling faster and more confident retrieval despite forget cues.

This resistance to forgetting was not specific to any particular emotional category. Rather, unexpectedness itself appeared to dominate emotional valence in determining memory persistence. This finding contrasts with reports that negative emotion alone can diminish DF effects (Hall et al., 2021; Nowicka et al., 2011), but it supports the broader idea that both surprise and emotion rely on overlapping neuromodulatory systems that bias encoding toward retention. The absence of a robust difference in DF between emotional and neutral items is consistent with the mixed nature of the existing literature. While some studies have reported reduced forgetting for emotional material, others have found comparable DF effects across emotional and neutral stimuli, suggesting that emotional modulation is context-dependent (e.g., Bailey & Chapman, 2012; Hall et al., 2021). One possible explanation is that emotional valence does not uniformly enhance encoding strength in a way that reliably disrupts forgetting processes. Instead, its effects may depend on factors such as arousal, attentional prioritisation, and task demands (e.g., Mather & Sutherland, 2011; Talmi, 2013). In the present context, unexpectedness may have served as a more potent cue for adaptive encoding than emotional arousal or valence, producing an across-the-board resilience to forgetting. This indicates that the expectation effect is robust across valence, but may not differ substantially in magnitude by emotion under the current paradigm. That said, the interaction between emotion and expectation for recollection (with weaker effects for positive stimuli) suggests the boundary may be subtler. Therefore, emotion can modulate the expectation advantage, and expectation modulates DF efficacy, but these factors do not always interact in a straightforward three-way manner (emotion × expectation × DF). A further consideration is that emotional valence also affected responses to new items. False alarms were more frequent for familiarity than recollection and were numerically higher for emotional than neutral foils, particularly negative foils. This pattern may reflect a more liberal tendency to classify emotional foils as old, but it may also reflect false familiarity arising from greater semantic, affective, or perceptual overlap among negative stimuli. The present design cannot distinguish decisively between these possibilities. Importantly, this issue relates primarily to valence effects on memory performance and it cannot explain the expectation effects identified here. This is because expectation status was defined only for studied items and false alarms were corrected separately by valence and response type.

The present findings also have implications for recent contextual accounts of DF, which propose that forgetting arises from shifts in mental context that reduce the overlap between encoding and retrieval (Sahakyan, 2024). From this perspective, processes that update internal context representations might be expected to facilitate forgetting. However, the current results show the opposite pattern: unexpected items, which are likely to induce prediction-error-driven context updating, were less susceptible to directed forgetting. One possible explanation is that expectation violation promotes the formation of more distinctive or strongly bound item–context representations, which remain accessible despite contextual shifts. In this sense, prediction error may both alter context and strengthen the encoded trace, with the latter reducing the effectiveness of forgetting. These findings therefore suggest that contextual mechanisms alone may be insufficient to account for DF effects, and that the strength and structure of encoding play a critical role in determining whether memories can be successfully downregulated.

In practical terms, this underscores that DF studies must consider not only the forget/remember instruction but also the encoding context—specifically, whether stimuli conform to or violate expectations. These findings also raise a critical theoretical question as to whether DF should be reconceptualised as a dynamic process contingent on encoding strength rather than a fixed instruction effect. This point is discussed further in the next section.

### Implications for Directed Forgetting and Memory Control

The current findings challenge the assumption that DF uniformly reflects voluntary, top-down control. Instead, they highlight that DF operates within a dynamic interplay between intentional regulation and stimulus-driven encoding. When bottom-up or extraneous processes, such as prediction error, are engaged, they can override or weaken intentional forgetting. This suggests that DF effectiveness depends on encoding strength and distinctiveness, and that surprise pushes memories beyond the suppression threshold. Furthermore, memory control may not be uniformly distributed across mnemonic subsystems but instead may depend on the representational depth of the memory trace, as revealed by the uneven effect of DF on recollection and familiarity. Recollection, dependent on hippocampal binding and contextual reinstatement (Kafkas et al., 2017; Montaldi & Mayes, 2010), appears more amenable to enhancement by expectation violation and less to inhibition by forgetting cues. Memory control may therefore operate differently across mnemonic expressions. Recollection, which depends on hippocampal binding and contextual reinstatement, was reduced by forget cues but was also the component most enhanced by expectation violation. Familiarity, in contrast, showed the reverse DF pattern, with higher familiarity-based recognition for TBF than TBR items. This suggests that forget cues may not eliminate memory traces uniformly, but may instead reduce access to contextual detail, shifting recognition from recollection towards familiarity. From a theoretical standpoint, these results place memory control within the broader predictive-processing framework. Expectation violation triggers a reallocation of attentional resources to support the updating of mental models (Frank & Kafkas, 2021). Such processes are adaptive in naturalistic contexts—helping organisms learn from prediction errors—but they also render memories more resistant to later suppression. DF may thus reveal a fundamental trade-off between adaptability and control. That is, the very mechanisms that ensure learning from unexpected events also reduce our capacity to deliberately forget them. Moreover, these findings have implications for individual differences in working memory capacity or inhibitory control, as well as cognitive ageing. Older adults with reduced inhibitory control might show even greater resistance to DF when stimuli are unexpected; similarly, populations with attentional-control deficits (e.g., ADHD) may show altered expectation × DF interactions. These speculations, therefore, require more direct exploration.

### Applied Implications for Memory Enhancement in Education

The resilience of unexpected information to forgetting has clear educational relevance. If surprise strengthens encoding and promotes recollection-based learning, then deliberate incorporation of expectation violations into instruction could improve knowledge retention. For example, introducing counterintuitive examples, challenging predictions, or highlighting anomalies within well-learned material may engage the prediction-error system, thereby promoting durable learning. Research on curiosity and adaptive memory supports this principle. Specifically, when learners’ expectations are violated, dopaminergic activity increases and enhances hippocampal encoding (Gruber et al., 2014; Kafkas, 2021). Educators might therefore benefit from designing lessons that strategically include unexpected or conceptually dissonant elements to elicit curiosity and deeper processing. However, these benefits should be applied judiciously. Overuse of surprise may fragment coherence or divert attention from core concepts. Moreover, because unexpected material appears less susceptible to deliberate forgetting, educators must ensure that such material aligns with learning goals, as erroneous or outdated information may then persist in memory. Nonetheless, integrating mild expectancy violations—through conceptual conflict, novel applications, or unpredictable testing formats—could enhance long-term retention and understanding more effectively than repetition alone.

### Applied Implications for Memory Control and Rehabilitation

The finding that unexpected events resist forgetting also holds implications for therapeutic and rehabilitative contexts where controlling memory strength is desirable. In clinical settings addressing trauma, addiction, or intrusive memories, interventions often rely on intentional suppression or extinction of unwanted memories (Anderson & Hanslmayr, 2014). The current data suggest that such strategies may be less effective for memories encoded under surprising or highly novel circumstances. Traumatic events, by their nature, are often unexpected and emotionally intense, thereby recruiting the same prediction-error and neuromodulatory mechanisms that enhance recollection. This dual engagement of surprise and emotion could partly explain the persistence of traumatic memories despite conscious attempts to forget.

Conversely, in rehabilitation or cognitive training contexts aiming to enhance retention, such as memory rehabilitation in ageing or neurological conditions, leveraging the effects of unexpectedness could be beneficial. Introducing mild prediction violations or novel associative pairings might improve learning outcomes by stimulating the same adaptive encoding pathways that resist forgetting. Such an approach aligns with evidence that novelty-based interventions can boost hippocampal plasticity and episodic memory in older adults (Steiger et al., 2023). Thus, understanding how expectation interacts with intentional control can inform both therapeutic forgetting and memory-enhancement strategies.

Taken together, these findings also have broader implications beyond specific applied domains. They also speak more broadly to the conditions under which memory can be successfully regulated. Specifically, the results suggest that the effectiveness of intentional forgetting depends critically on encoding strength and representational distinctiveness, providing a constraint that applies across domains. In this sense, similar challenges may arise in both educational and clinical contexts, where memories formed under conditions of high salience or unpredictability may be more difficult to downregulate. This has implications for domains such as eyewitness memory, where unexpected or emotionally salient events may be preferentially retained and less susceptible to intentional forgetting. The present findings therefore highlight that attempts to modulate memory after encoding may be limited when initial encoding processes have produced strong or distinctive representations.

### Limitations and Future Directions

While the current study provides compelling evidence for the resistance of unexpected events to DF, several limitations should be acknowledged. Firstly, the emotional stimuli differed in arousal as well as valence. This reflects a common challenge in emotional memory research, where valence and arousal are often closely linked and difficult to dissociate (e.g., Mather & Sutherland, 2011; Talmi, 2013). Indeed, both dimensions have been shown to contribute to memory performance, and their relative influence may vary depending on task demands and stimulus characteristics (Gao et al., 2024; Kensinger, 2009). As a result, the present findings cannot disentangle the relative contributions of valence and arousal. However, additional analyses including condition-level arousal ratings did not alter the pattern of results, suggesting that the findings are not solely driven by variation in arousal. Nevertheless, future work should orthogonally manipulate these dimensions to clarify their interaction with expectation and forgetting (for this see Mukhtar & Kafkas, 2026). Our sample comprised exclusively healthy young adults. Therefore, extending this research to older adults or clinical populations with altered inhibitory control will extend the generalisability of the findings or further clarify their limits. Furthermore, comparing item-method DF (as used here) with list-method DF and retrieval-based suppression tasks (e.g., think/no-think) would reveal whether resistance to forgetting under unexpectedness generalises across different forms of memory control. Finally, future studies should combine this paradigm with neuroimaging or electrophysiological indices of prediction error, such as midbrain or hippocampal activation and pupil dilation (Kafkas, 2021, 2024), to explore the neural bases of these effects and explore hypotheses related to the effectiveness of memory control under different experimental conditions.

## Conclusion

The present study demonstrates that expectation violations confer a robust recollection advantage and render recollection-based memories more resistant to intentional forgetting. Emotion modulates, but does not eliminate, this effect. Specifically, the impact of surprise persists across valence categories, although it is attenuated for positive stimuli. These findings challenge models of directed forgetting that view memory suppression as a purely top-down process, instead situating it within a predictive-processing framework in which encoding strength and surprise constrain control. Beyond theoretical relevance, the results suggest that harnessing moderate expectancy violations can enhance learning, while understanding their resistance to suppression can inform clinical approaches to memory regulation. The interplay between expectation, emotion, and cognitive control thus defines a fundamental balance between the flexibility to learn from the unexpected and the ability to forget what no longer serves us.

## Data Availability

All data and materials will be made available from the corresponding author upon request.
